# Closed posterior dislocation of the ankle without fracture

**DOI:** 10.4103/0019-5413.41864

**Published:** 2008

**Authors:** Alok C Agrawal, HKT Raza, RU Haq

**Affiliations:** Department of Orthopedics and Traumatology, Netaji Subhash Chandra Bose Medical College and Hospital, Jabalpur - 482 003, MP, India

**Keywords:** Closed injury, posterior dislocation ankle

## Abstract

Closed posterior dislocation of the ankle without a fracture is a rare injury. We are reporting a case in a young male on his motorbike, being hit at the right ankle from behind. The mechanisms of injury along with MRI findings are being discussed.

## INTRODUCTION

Dislocation of the ankle without associated fracture or wound is an extremely rare injury.^1^ Fahey and Murphy^2^ classified tibio-talar dislocations into anterior, posterior, medial, lateral, superior or combinations of these basic displacements. Most of these are either open and/or with an associated fracture of the tibia, fibula or the talus itself. Of these the posterior-medial dislocation has been described most often in the literature.^1^–^5^ Most authors have described this injury in young adult males. Falls, road traffic accidents and sports have been described as the most frequent causes of these injuries. Forced inversion or eversion with axial loading in a maximally planter-flexed foot is thought to be the cause of this injury. The patho-anatomy of this injury has been dependent on findings during surgical repairs and has not been described accurately. We are reporting a case of closed posterior dislocation of the ankle without fracture in an 18-year-old male patient following road traffic accident. The most probable mechanism is forced forward displacement of the tibia leaving the talus behind. The patho-anatomy as evident from the magnetic resonance finding is also being described. The injury is being described not only for its rarity but also to discuss its unique patho-mechanics, mechanism of trauma and its prevention.

## CASE HISTORY

An 18-year-old, 6 feet 4 inch tall male weighing 90 kg presented with pain, swelling and deformity one hour after a road traffic accident. The patient was on a bike when he was hit from behind over the right leg just above the ankle by another fast-moving vehicle where the large heel-breast of his shoes got stuck in the footrest and the leg was pushed anteriorly with great force resulting in a closed posterior dislocation of the talus from the ankle mortise. Physical examination revealed a deformed ankle with foot posteriorly displaced. There was no open injury. Swelling was present. The dorsalis-pedis and posterior tibial pulsations were normal. There was no hypoaesthesia, hyperlaxity or associated injuries.

**Figure 1:**
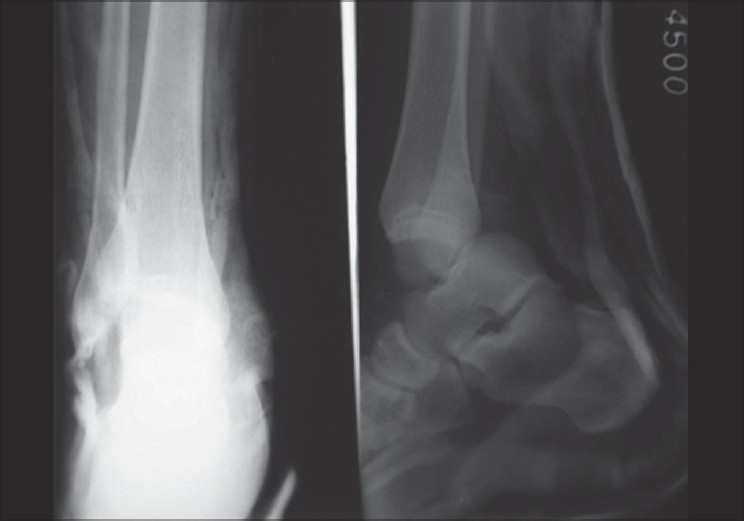
AP and lateral X-ray of right ankle shows posterior dislocation of the ankle

**Figure 2:**
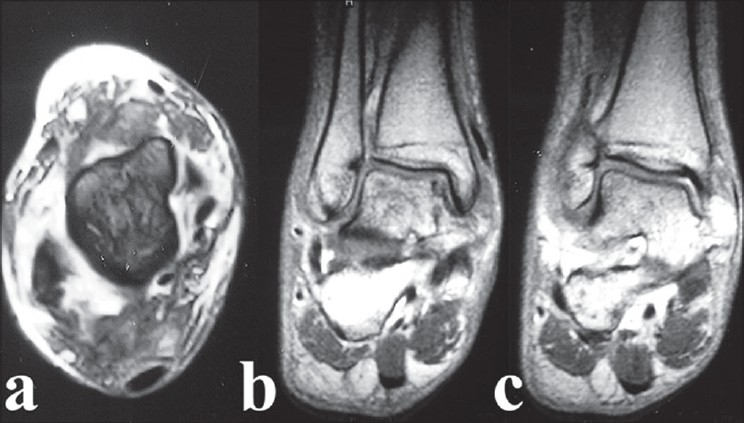
(a) GRE T2 Weighted image at the level of the talar dome revealed edema in the soft tissues anteriorly but no talofibular ligament is evident due to tear. (b) T1 weighted coronal image at the level of sustantaculum -tali reveals a tear of the medial collateral ligament. (c) T1 weighted coronal image. The joint between the sustantaculumtali and the talus is absent and is replaced by a thin hypointense line suggestive of a fibrous tarsal coalition

**Figure 3:**
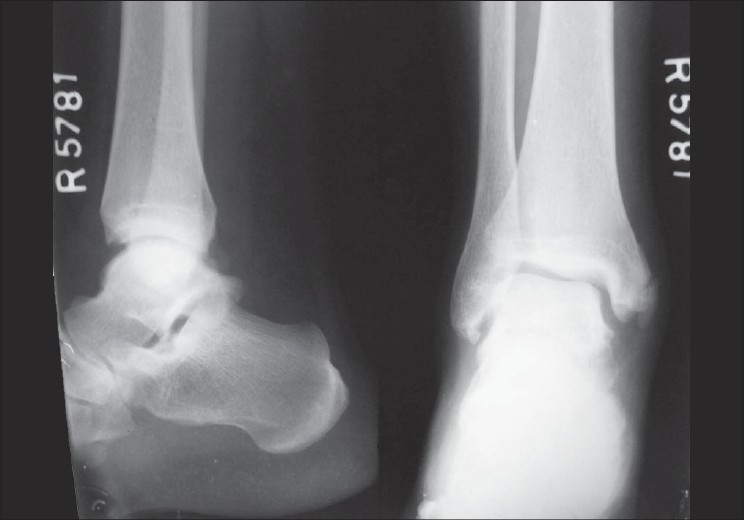
Follow-up X-ray of the ankle revealing osteophytes, calcification of the collateral ligaments beneath the malleoli with mild subluxation of the ankle joint

Plain anterior-posterior and lateral radiograph of the right ankle demonstrated a posterior dislocation of the ankle without any fracture or widening of the tibio-fibular syndesmosis [Figure 1]. Patient was treated by leg elevation, above knee slab application and analgesics followed by closed reduction under general anesthesia and application of an above knee cast. Post reduction magnetic resonance imaging [Figure 2] demonstrated a torn anterior talo-fibular ligament and medial collateral ligament. A fibrous talo-calcaneal coalition was also found. He was advised surgical repair of the ligaments which he refused following which he was advised not to bear weight for six weeks. On follow-up at two years although he had painless normal range of ankle motion with full weight bearing and squatting, the x-ray of the ankle revealed osteophytes, calcification of the collateral ligaments beneath the malleoli with mild subluxation of the ankle joint [Figure 3].

## Discussion

Dislocation of the ankle requires considerable force because of the mechanical efficiency of the mortise and the strength of the associated ligaments.^3^ Since ligaments are stronger than the malleoli, most ankle dislocations are associated with fractures. Wilson *et al.*, reviewed the literature prior to 1939 and found 16 cases of ankle dislocation without fracture.^1^ More recently Soyer *et al.*, (1994) found 73 cases in the relevant literature.^4^ About 50% of ankle dislocations are usually open. However, in our case there was no open injury. An increased participation in outdoor activities is probably the cause of the higher incidence of this injury in young males. Our patient was also an 18-year-old, strong, adult male. Unlike ankle sprains, which predominantly occur in sportsmen, ankle dislocation is caused mainly by road traffic accidents, particularly motorcycle accidents. Sports trauma is the second most common cause.

**Figure 4:**
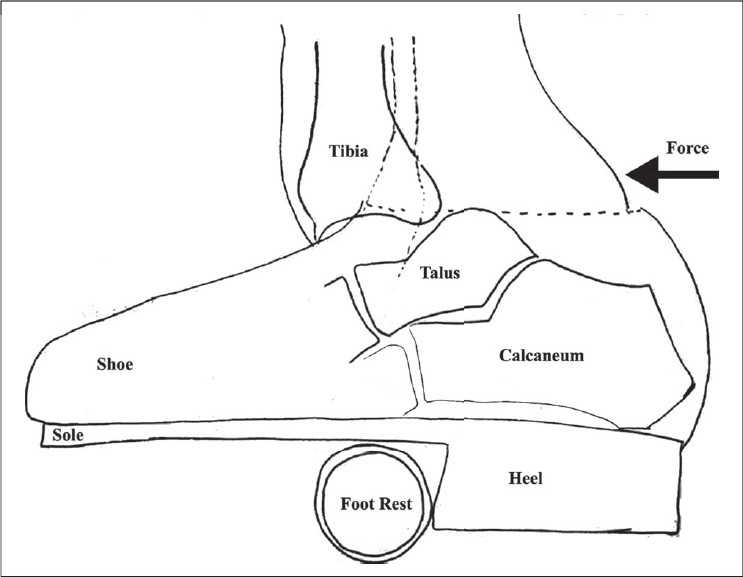
Line diagram shows patient's foot resting on footrest of motorcycle. The large heel of the shoe getting stuck. The force of motorcycle wheel from behind has dislocated the ankle without fracture

The exact patho-mechanics of this injury has not been described accurately. Most authors suggest the cause as a combination of inversion along with axial loading while the foot is maximally plantar-flexed. This hypothesis is supported by experimental work done by Fernandez^5^ on cadavers. The ligaments, which he found to be injured in this type of injury, were the anterior talofibular and calcaneofibular ligament. He also postulated that once the ankle is dislocated without fracture, the tendon of calcaneus pulls it posteriorly. Most authors have supported this postulate. However, Wroble *et al.*, were of the opinion that dislocations of the talus occur because of extrusion of the talus anteriorly or posteriorly when force is applied in a plantar-flexed foot.^6^ In our case, the patient was wearing large shoes resting on the footrest well supported on it by the high heel-breast of the shoe. Being hit from behind above the ankle the patient's foot being plantar-flexed at this time got stuck at the footrest due to the high heel-breast, resulting in the tibia being forcefully pushed anteriorly, leaving behind the talus and the foot [Figure 4]. The associated talocalcaneal bar prevented an associated subtalar dislocation.

Since the injury occurs due to inversion, the structures that are primarily torn are the anterior talofibular, the calcaneofibular and posterior talofibular respectively. The deltoid ligament is usually spared. However, in our case since the mechanism of injury was not inversion with axial loading but a forward extrusion of the tibia leaving the talocalcaneal complex with the foot behind, we expected both medial and lateral collateral ligament injuries. Our clinical suspicion was confirmed by MRI report, which documents complete tear of the anterior talofibular and medial collateral ligament. Interestingly, our patient had a talocalcaneal coalition. This may have predisposed the patient to have an ankle dislocation rather than a subtalar dislocation when he was hit from behind. Tarsal coalition as a predisposing cause of ankle dislocation without fracture has not been previously described in the literature.

We are reporting this case for its unique mechanism of injury, MRI findings and outcome. We recommend that for racing bikes the footwear should not have a heel with a high breast-line; preferably, they should have a flat sole which will not permit the heel getting stuck in an accident, bringing about this type of grave injury.
